# COVID-19 Isolation in Healthy Population in Israel: Challenges in Daily Life, Mental Health, Resilience, and Quality of Life

**DOI:** 10.3390/ijerph18030999

**Published:** 2021-01-23

**Authors:** Lena Lipskaya-Velikovsky

**Affiliations:** School of Health Profession, Sackler Faculty of Medicine, Tel Aviv University, Tel Aviv 69978, Israel; lenasky@gmail.com; Tel.: +972-3640-5442

**Keywords:** secondary pandemic impact, lockdown, psychological distress, participation in daily life activities, loneliness

## Abstract

Background: Pandemics produce long-lasting secondary impacts on health, with a significant burden on people and society. Until now, the secondary impact of COVID-19 has been little estimated. Our aim was to investigate factors underlying quality of life (QOL) during COVID-19 lockdown among a healthy population, while QOL reduction expands vulnerability to the pandemic secondary impact. Methods: During the spring lockdown in Israel, 571 healthy adults completed a survey that included standard measurements for psychological distress, participation in daily life activities, a sense of social connectedness, resilience, and QOL. Results: We found a high level of psychological distress, significant reduction in participation dimensions, and in QOL (psychical, psychological, and social). These indices were even lower among women, younger adults, and the unemployed. Path analysis demonstrated that psychological distress, participation dimensions, social connectedness, and self-efficacy explained QOL, while participation dimensions were found to be the mediators. Conclusions: The COVID-19 has had a wide impact on the general population, with the potential for negative secondary impacts. Women, young adults, and the unemployed are at high risk for secondary effects. Public health strategies should address the reported factors and populations in order to improve QOL in a healthy population and limit the impact of the pandemic.

## 1. Introduction

The coronavirus (COVID-19), with its high contagiousness and exponentially increasing incidence of infection, has posed many challenges to health-care systems and policymakers. Due to the complexity of primary coping with COVID-19, the “indirect effect” or “secondary impact” of the pandemic on the population has yet to be widely addressed [1,2,3,4]. The secondary impact of the pandemic on health includes the development of aversive health conditions that are not directly related to the primary infection, rather are provoked by the situation, such as mental health conditions. In addition, the existing health conditions are deteriorating due to both the pandemic and constraints of health services, which are mainly directed to cope with it [2,4,5]. Unfortunately, it was demonstrated that the secondary impact of the epidemic can last longer than the epidemic itself, has greater prevalence, and generates high burdens on people and the whole society due to increased level of disability and extensive post-pandemic usage of health and social services [2,5,6,7].

There are multiple mechanisms of the pandemic secondary impact on health and health-related quality of life (QOL). Health-related QOL is a subjective, multidimensional concept, which emphasizes the importance of self-perception of individuals’ current state in life in the context of culture and value systems in which they live, and in relation to their goals, expectations, standards, and concerns. QOL incorporates individuals’ perception of physical health, psychological state, level of independence in daily life, social relationships, personal beliefs, and their relationship to salient features of the environment [8,9]. Indeed, it was found that QOL adds meaningful information beyond traditional biomedical factors to the prediction of objective health factors (such as illness severity and mortality) and/or usage of intensive health care services in the short- and long-term [10]. 

QOL during a pandemic may be affected by dramatic changes in personal routines with alternating patterns of some everyday life activities and aborting others, challenging personal standards, expectations, and experience of independence and competence in daily life [11,12]. These changes happen due to the pandemic itself or coping strategies such as social distancing and lockdown distorting objective and subjective aspects of social interactions, leisure and sports activities, work and school arrangements, and even sleep patterns and home-management [11,12,13]. Alteration in daily life activities out of personal control should raise thorough concern since through their occupation, people are able to meet their needs and bring meaning to their lives, express themselves, communicate with others, and develop their skills [8,14]. Health damages due to restricted participation were previously demonstrated in various life situations, such as emigration or marginalization [15,16,17,18] and were initially reported in COVID-19 [19,20]. 

The secondary impact of a pandemic may be driven by a psychological mechanism [12,21], which is a substantial component of QOL. The psychological impact of pandemics includes a variety of signs of anxiety, pressure, depression, fear, confusion, anger, and even post-traumatic stress symptoms, all of which affect the overall mental health of a population [4,11,22]. Psychological signs and conditions may be a direct result of a pandemic itself (e.g., anxiety and fear for oneself and others’ health), or they may be a result of the general situation due to a pandemic (e.g., stress due to employment status and economic uncertainty) [7,12,21,23]. It was suggested that the longer the situation persists and the less adequate the services and supplies become, the higher the psychological impact [22]. Indeed, a high level of psychological distress due to COVID-19 was already demonstrated in China and Japan [7,23]. However, it was less investigated in Israel.

COVID-19 coping strategies of social distancing, lockdown, and managing social interaction through technology instead of physical closeness may further contribute to the pandemic’s impact on the aspect of the social relationship of QOL, resulting in strong feelings of social isolation and loneliness. Previous studies demonstrated that loneliness has an adverse impact on physical and mental health, including depression and poor sleep quality [24,25,26]. Ironically, it was found that people reporting a higher level of social isolation had an unfavorable cardiovascular function and impaired immune system functioning [26,27], all of which are hazardous complications of COVID-19. The situation may be even more complicated, producing an even higher decline in health and QOL, since loneliness and changes in daily life activities may lead to cognitive decline (e.g., global cognitive function, memory, and executive functions), further limiting resources for coping with a rapidly changing situation out of one’s personal control [24,26,28,29]. On the other hand, factors such as resilience and self-efficacy, which refer to personal beliefs aspect of QOL and are found to be a strong positive predictor of QOL, may have a protective effect helping to cope with challenging life conditions and reducing the impact of pandemic [30]. 

To summarize, multiple factors resulting from COVID-19 outbreak have a high potential to reduce current health-related QOL, which was found to be a strong predictor of long-term parameters of health and well-being [10,12,21]. However, the impact of COVID-19 outbreak and following lockdown on QOL of a healthy, not-infected population was less investigated in Israel. Further investigation of the factors that have high potential to generate secondary impact at the population level will be informative for building effective health promoting strategies for those who did not acquire the infection or were cured from the virus [1,2]. Thus, the aim of the study was to investigate factors underlying QOL during COVID-19 lockdown among a healthy population since QOL reduction expands vulnerability to the pandemic secondary impact. More specifically, we addressed psychological distress, objective and subjective dimensions of participation, resilience through self-efficacy, and social connectedness among the healthy population in Israel during the Spring COVID-19 lockdown and their impact on health-related QOL. We hypothesized that a high level of psychological distress and loneliness, as well as restrictions in daily life activities, will characterize the healthy population during the lockdown, and all of them will lead to a decrease in health-related QOL.

## 2. Materials and Methods

This is a cross-sectional study with a convenience sample that was conducted by means of an internet survey during the Spring 2020 COVID-19 lockdown in Israel. The study was approved by the Ethics Institutional Committee according to the Helsinki Declaration. Healthy participants, by their report, without neurological, mental, or physical disability, not infected by the COVID-19 virus, and aged 18 and older, were reached using social networks. The participants provided informed written consent, and those who met the study criteria received the survey containing the following instruments. We used the World Health Organization Quality of Life Instrument (WHOQOL-BREF) to evaluate 4 domains of health-related quality of life: Physical, psychological, social, and environment, based on 26 statements [9]. The Adults Subjective Assessment of participation (ASAP) was used to evaluate 5 dimensions of participation in 52 daily activities: Participation diversity (number of participated activities), number of stopped activities (due to COVID-19 regulations), participation intensity (frequency), with whom the activities were done, where they were done, satisfaction with participation, enjoyment, and experience of meaning [31]. The Depression Anxiety Stress Scale (DASS-21), the 21-items’ version was used to estimate psychological distress [32]. The Revised U.C.L.A. Loneliness Scale [33] of 20 items was used to assess levels of loneliness. The level of community connectedness was evaluated using 24 items Sense of Community Index, version 2 (SCI-2) in 4 aspects: Reinforcement of needs, membership, influence, and shared emotional connection [34]. Self-efficacy was evaluated with a General Self-Efficacy Scale of 14 items [35]. The survey was completed by 571 people, including all the tools. 

Descriptive statistics were used to characterize the participants and main measurements. The type of distribution was detected using the Kolmogorov–Smirnov test. To estimate between-group differences, we used an independent samples *t*-test and one-way ANOVA or Mann–Whitney and Kruskal–Wallis tests, depends on the type of distribution, with correction for multiple comparisons. For each aspect of QOL, a multivariate model of explanation was built, using linear regression with a stepwise method. Based on the preliminary analysis, the following parameters were entered into 4 regression models: GSE, anxiety, stress, and depression subscales of the DASS, UCLA score, 4 sub-scales of SCI, experience of meaning in participation, satisfaction with participation and enjoyment, age, gender, and employment status. In addition, the model for explanation of the physical and environment domain included a number of stopped activities; the model of psychological domain included participation diversity; the model of social relationship domain included participation intensity, and percent of activities done alone. Finally, we entered into the structural equation model variables that were found explaining in the best way the QOL dimensions based on the regression analysis. It was estimated whether relevant participation dimensions mediated the relationship of other explanatory variables (psychological distress, social connectedness, self-efficacy, and demographic data) with the QOL dimensions. The following criteria were used to evaluate fitting of the models to the data: (1) A non-significant Chi-square *p*-value (*p* > 0.05); (2) a comparative fit index (CFI) value greater than 0.90; (3) a Tucker–Lewis Index (TLI) value greater than 0.90; and (4) a root mean square error of approximation (RMSEA) value less than 0.05. We reported on the mediation effect of the variables based on regression weight and standard error (SE) (*p* < 0.05). The data were analyzed using SPSS (IBM, Version 25, Armonk, NY, USA) with an additional AMOS (IBM) package for mediation effect analysis. The level of significance was set at 0.05.

## 3. Results

### 3.1. Descriptive Analysis 

The survey was completed by 441 women (77.2%) and 129 men (22.6%), aged 18–83 (Mdn = 29; IQR: 25–46). More than half of participants had a spouse (*n* = 360, 63%), lived with families started by them (*n* = 354, 62%), and almost half of them had children (*n* = 240, 42%). Most of the participants lived in an urban area (*n* = 421, 73.7%), and about half of all the study participants lived in the geographic center of the country (*n* = 309, 54.1%). During the COVID-19 lockdown period, 274 participants (48%) had been working, and 218 (38.2%) did that from home. Among those who did not work during the lockdown, 18 participants (3.15%) were retired, 134 (23.45%) were unemployed before the COVID-19 outbreak, and 145 (25.4%) were not working due to the lockdown. Only 94 participants (16.5%) were in the risk group, which was defined for the purpose of this study as the following: People aged 60 or older, or people with cardiovascular conditions (e.g., high blood pressure), or respiratory conditions (e.g., asthma), or immune system impairments and conditions (e.g., diabetes) or heavy smokers (>20 cigarettes per day), based on self-report. During the period of the report, 469 participants (82.1%) tried to keep daily routines during lockdown and 392 (68.7%) reported on going out for their errands and chores during the previous 3 days. More than half of the participants (*n* = 338, 59.2%) reported on managing interpersonal communication mainly in a remote way. Fifty percent of the participants reported that they stopped participating in about 16 activities during lockdown (Mdn = 16, IQR: 13–20). Half of the participants engaged at that period in 13 activities or less (Mdn = 13, IQR: 11–15), performing them with a frequency of one up to three times a week (Mdn = 4.4, IQR: 4–4.75). The participants reported on some level of enjoyment with their activities (Mdn = 4.4, IQR: 4–4.8) and reported on moderate meaning (Mdn = 242 (of 390 for the calculated Mdn), IQR: 187–293), being somewhat satisfied with their activities (Mdn = 4.7, IQR: 4.3–5.1). Half of the participants performed 83.3% or more of their activities at home (Mdn = 83.3, IQR: 69.4–100%) and 51.4% or more of their activities were performed alone (Mdn = 51.4, IQR: 33.3–70.2%). In addition, more than half of the participants reported that they communicated with a different social milieu during the lockdown than in their everyday life (*n* = 335, 58.7%) and managed their communication mostly through technological devices (*n* = 338, 59.3%). 

Among the study participants, only 219 (38.4%) reported on a normal level of stress, 302 (52.9%) on normal level of anxiety, and 271 (47.5%) normal level of depression. Around a quarter of the participants reported on extremely high levels of psychological distress (stress: *n* = 176, 30.8%; anxiety: *n* = 129, 22.6%; depression: *n* = 137, 24%). During the COVID-19 lockdown, the following parameters of QOL were reported for physical domain: Mdn = 69, IQR: 56–81; psychological domain: Mdn = 69, IQR: 63–81; social relationship domain: Mdn = 75, IQR: 50–81; environment domain: Mdn = 81, IQR: 69–88.

### 3.2. Analysis of Differences

**Figure 1:**
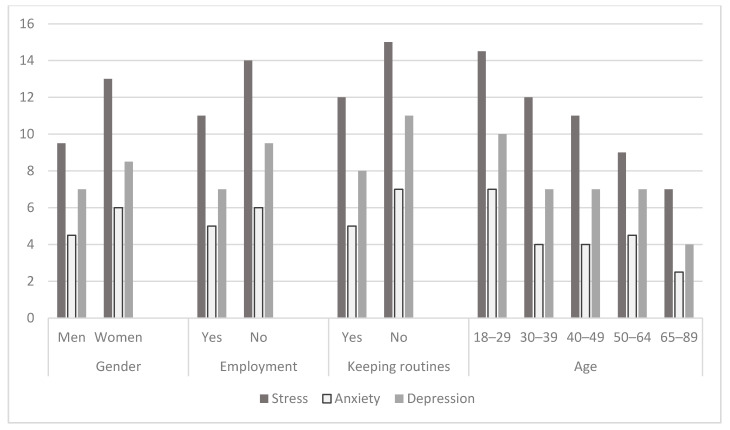
Psychological distress by groups—The Depression Anxiety and Stress Scale (*n* = 571).

**Figure 2:**
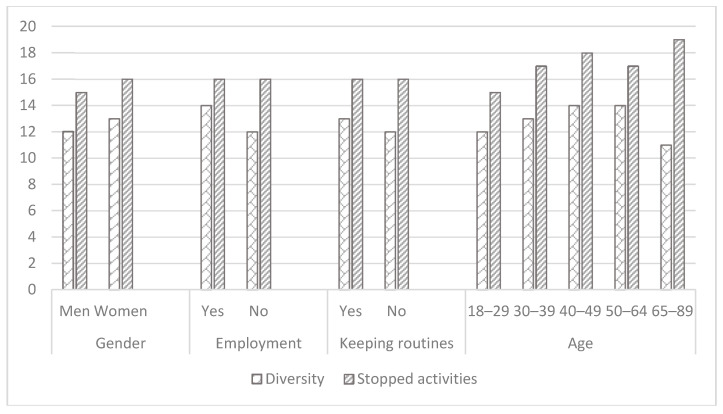

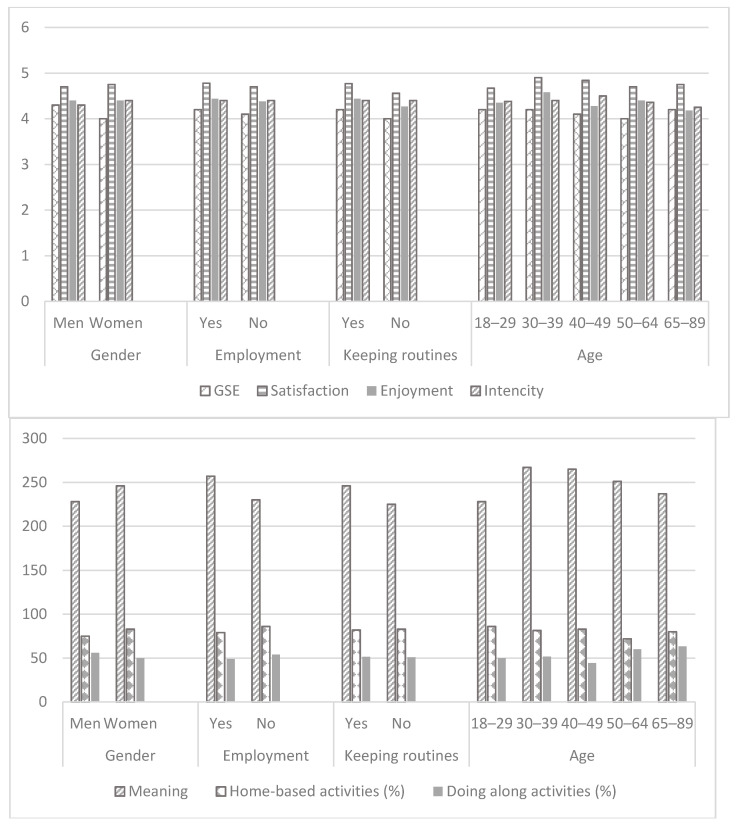
Participation dimensions (Adults Subjective Assessment of Participation) and self-efficacy by the groups (*n* = 571).

**Figure 3:**
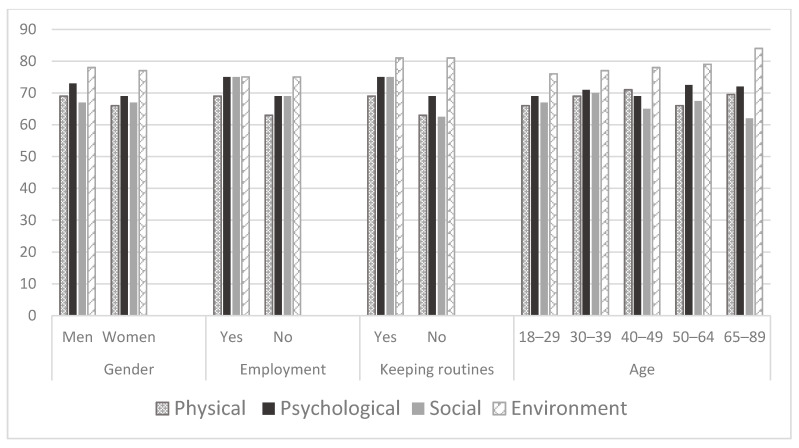
Quality of life domains by groups—WHOQOL-BREF (*n* = 571). Note: WHOQOL-BREF—WHO Quality of Life instrument.

We investigated differences in main study variables by gender, employment status, age, health conditions, manner of communication, and daily life routines. Women reported a higher level of stress, anxiety, and depression than men (Table 1, Figure 1). Following the COVID-19 lockdown, they stopped participation in more activities, still being engaged in more activities than men. They performed more activities at home, while reported on being less engaged in occupations alone, and had a higher experience of meaning in occupations (Table 1, Figure 2). Still, women reported on lower parameters of QOL in physical and psychological domains (WHOQOL-BREF) (Table 1, Figure 3).

**Figure 4:**
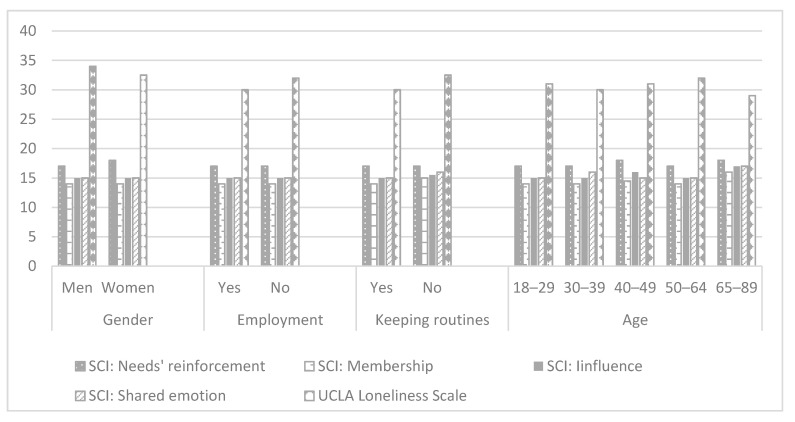
Social and community connectedness (*n* = 571). Note: SCI—Sense of Community Index.

Employed participants reported on significantly lower signs of stress, anxiety, and depression (Table 1, Figure 1), lower levels of loneliness (Table 1, Figure 4), higher self-efficacy, and higher parameters of participation in the following dimensions: Participation diversity, meaning, satisfaction, percent of activities done at home and percent of activities done alone (Table 1, Figure 2). Moreover, working participants reported a significantly higher QOL in physical, psychological, social, and environmental domains (Figure 3). 

Participants belonging to the risk group for consequences of COVID−19 reported a significantly higher percentage of activities done alone than those who were not at risk. The at-risk group experienced a lower level of stress during lockdown than those who were not at risk, while still less enjoyed participating in their activities (Table 1, Figure 1, Figure 2, Figure 3 and Figure 4).

Participants who communicated mostly through the technological means reported on a higher percent of activities that were done alone (through technological means: Mdn = 53.8, IQR: 35.3–72.8; directly: Mdn = 50, IQR: 32–68) in comparison to those who mostly communicated directly (Table 1). Still, the latter reported higher QOL in the physical domain (through technological means: Mdn = 63, IQR: 56–81; directly: Mdn = 69, IQR: 56–81) (Table 1).

Even though the age groups were not equal as for the number of the subjects (18–29: *n* = 288, 50.4%; 30–39: *n* = 110, 19.3%; 40–49: *n* = 44, 7.7%; 50–64: *n* = 112, 19.6%; 65–85: *n* = 17, 3%) and the results may be less indicative, still, the findings demonstrate that participants in the youngest group (aged 18–29) experienced a higher level of stress, anxiety, and depression during lockdown than the older ones (Table 1, Figure 1). Differences were found in the number of participated activities, the number of stopped activities, the experience of meaning in participation, enjoyment with participation, percent of activities done at home, percent of activities done alone, due to differences between those in their 30s’ and those who in their 20s’ and 50−60s’ in favor of those who aged 30s’ (Table 1, Figure 2). In addition, differences were found in the environmental domain of QOL. The lowest parameters were found among the youngest participants (Table 1, Figure 3).

Participants who reported trying to keep daily routines during the lockdown reported a significantly lower level of stress, depression (Table 1, Figure 1), and loneliness (Table 1, Figure 4), significantly higher general self-efficacy, participation diversity, meaning in participation, enjoyment from participation, and satisfaction in comparison to those who were not keeping daily routines during the lockdown (Table 1, Figure 2). In addition, participants keeping daily routines reported higher physical, psychological, and social domains of QOL (Table 1, Figure 3). Since keeping daily routines during lockdown was found to be contributing to the main research parameters, we explored the interaction effect between this variable and additional demographic variables of gender, age, and employment status. The effect of interaction between gender and keeping daily routines was found on anxiety (F(3) = 8.1, *p* = 0.005), UCLA loneliness scale (F(3) = 9.95, *p* = 0.002), and Social domain of QOL (F(3) = 4.6, *p* = 0.032). A higher level of anxiety and loneliness and lower QOL were found among men who did not keep daily routines, in comparison to other groups. In addition, we found the effect of interaction between keeping daily routines and age on the Sense of Community Index subscales: Reinforcement of needs (F(4) = 2.54, *p* = 0.039), membership (F(4) = 2.76, *p* = 0.027), influence (F(4) = 3.84, *p* = 0.004), and shared emotional connection (F(4) = 3.22, *p* = 0.013). Participants aged 40–49 who kept daily routines experienced significantly higher connectedness to the community than participants of the same age group who did not keep the routines during the lockdown. No additional effects of the interaction were found. 

### 3.3. Correlational Analysis

Higher QOL was strongly associated with lower levels of stress, anxiety, and depression, the experience of loneliness, a sense of belonging, and high self-efficacy (Table 2). In addition, higher QOL was weakly to moderately correlated with dimensions of participation, mainly the subjective ones, such as satisfaction with participation, enjoyment, and experience of meaning in participation, as well as with the objective dimensions of participation diversity and the number of stopped activities (Table 2). Furthermore, higher subjective participation dimensions of enjoyment, satisfaction, and meaning in occupations were associated with lower levels of stress, anxiety, and depression, with lower experience of loneliness and a higher sense of belonging based on SCI (Table 2). The objective participation dimension of the number of stopped activities was correlated with a sense of belonging, and percent of activities done alone was correlated with the experience of loneliness (Table 2).

### 3.4. Regression Analysis

It was found that the physical domain of QOL was explained by the level of stress and depression, number of stopped activities, enjoyment with participation, general self-efficacy, and influence sub-scale of SCI (F(6563) = 44.2, *p* < 0.001; R2 = 32) accounting for 32% of variance. The most contributing variable for the explanation was the level of stress, followed by the level of depression and the extent of enjoyment from participation (Table 3). The psychological domain of QOL was explained by levels of depression and stress, general self-efficacy, satisfaction with participation and membership sub-scale of SCI (F(5564) = 163.57, *p* < 0.001; R2 = 0.592), accounting for 59.2% of variance, with the highest contribution of the level of depression, followed by self-efficacy and satisfaction (Table 3). The social relationship domain of QOL was explained by levels of depression and stress, the experience of loneliness, satisfaction with participation, and percent of activities that were done alone (F(5564) = 69.78, *p* < 0.001; R2 = 0.382) accounting for 38.2% of the variance. The most contributing variable was the experience of loneliness, followed by satisfaction with participation and level of stress (Table 3). The environment domain of QOL was best explained by levels of depression and stress, the experience of loneliness and general self-efficacy, membership sub-scale of SCI, number of stopped activities, and age (F(7562) = 43.47, *p* < 0.001; R2 = 0.351) all together accounting for 35.1% of variance. The most contributing variable was general self-efficacy, followed by the experience of loneliness and depression. 

### 3.5. Mediation Analysis

Confirmatory path analysis demonstrated that the number of stopped activities and enjoyment with participation mediated the effect of general self-efficacy, sense of community belonging, and psychological distress (number of stopped activities mediated stress and enjoyment mediated depression) on the physical domain of QOL (Table 4). Satisfaction with participation mediated the effect of depression, general self-efficacy, and sense of community belonging on the psychological QOL domain and the effect of depression and experience of loneliness on the social relationship QOL domain (Table 4). In addition, the effect of depression, stress, and loneliness on the social domain was mediated by the percent of activities done alone (Table 4). The effect of stress, general self-efficacy, sense of community belonging, and age on the environment domain of QOL was mediated by the number of stopped activities (Table 4).

## 4. Discussion

The pandemic outbreak has the potential to produce a secondary impact on a range of health parameters and QOL of a not-infected, overall healthy population for even longer periods and with a higher burden than the virus itself [2,5,6,7]. This study demonstrated that the COVID-19 lockdown affected QOL, generated a high level of psychological distress and the experience of loneliness, limited participation in daily life and sense of belonging, while the impact of all these factors were deeply interwoven. 

### 4.1. Psychological Distress and Loneliness

Similarly to recent reports from China and Japan [7,23], coping with COVID-19 and the lockdown in Israel led to a high level of psychological distress. These findings further contribute to the existing literature on the effect of epidemics on mental health at the population level [4,11,12,21,22]. Moreover, the association of level of stress, anxiety, and depression with all domains of QOL suggests that psychological distress may affect health parameters, resulting in additional burden [2,5,6,7]. 

Following previous literature [35,36], our study showed that women, and unemployed, but also those in their 20′s with a lower level of contagion and, thus, at a lower priority for the actions of health services, tended to experience a higher level of psychological distress. These findings indicate that lockdown puts people who were not at risk for immediate COVID-19 impact to be at risk for the secondary impact of COVID-19. 

Interestingly, those who belong to a risk group for COVID-19 were in a better position with their psychological functions during the lockdown, reflecting the importance of the experience of meeting needs for psychological wellbeing. We surmise that people belonging to the risk group were naturally oriented toward keeping themselves safe and healthy, therefore, they viewed their lockdown as positive. Whereas those not at risk were more heavily concerned about other life aspects, e.g., keeping a source of income [12,21]. 

As was expected, COVID-19 regulations for physical distance and lockdown interfere with the experience of social connectedness. People experienced a higher level of loneliness when they communicated mostly with a different social milieu than they used to before COVID-19. An in-depth understanding of the social communication patterns and related factors is of importance due to their contribution to psychological wellness, QOL, and health [25]. For example, we found that employment contributed to a higher sense of connectedness and higher QOL, emphasizing the importance of keeping work as a resource for health [37]. Another protective factor against the experience of loneliness was keeping daily routines, which reflects the beneficial nature of daily life activities as enablers for connectedness and mental health [14]. Interestingly, new modes for managing social interaction, such as remote ones, contributed to the experience of social connectedness similarly to face-to-face interaction, suggesting that mass usage of technology that started with the COVID-19 outbreak has been beneficial during the lockdown. 

### 4.2. Participation in Daily Life

Lockdown and physical distancing dramatically changed people’s patterns of participation, raising great concern. The findings demonstrate that at least half of the participants had discontinued a high number of activities while only a limited range of activities was maintained. As expected, among the study participants, the locus of the participation was at home, and involvement of other people in the activities was limited, the conditions that were previously found to be restrictive for mental health [4,19]. Moreover, at that period, the participants ascribed quite low meaning to the activities that provided only moderate enjoyment. Still, they experienced satisfaction from the performance of their activities. These changes in participation patterns were produced rapidly by the situation and diminished the health benefits of participation, thereby affecting health and well-being [15,16,17,18]. 

However, the impact of lockdown on participation patterns was depending on multiple factors, such as age and social roles. Again, women, unemployed participants, and those in their 20′s or 50′s and older experienced the highest impact of lockdown on their participation patterns. Unfortunately, the first three populations were those who reported on higher psychological distress and lower QOL, further demonstrating the contribution of the participation to mental health and QOL, but also indicating the vulnerability of these populations. Although the pandemic had a direct impact on most objective participation dimensions, the number of stopped activities, representing the extent of the impact of the pandemic on a person’s life, was mainly found to be associated with psychological distress and QOL domains. In addition, we found that higher experience of meaning, satisfaction, and enjoyment from participation in maintained activities have been associated with a lower level of psychological distress, higher resilience, and QOL, having the potential to mitigate the impact of the pandemic. 

### 4.3. Quality of Life

The main focus of this study was health-related QOL as a predictor of long term health and well-being parameters [9]. During the spring lockdown in Israel, about half of the participants reported a low QOL in physical, psychological, and social relationship domains [38]. These findings can be easily understood in light of the general circumstances of threat to health and multiple social and physical restrictions during the lockdown. However, an in-depth analysis reveals unexpected findings. Interestingly, the environment QOL domain was little affected by the situation. As this domain addresses, among others, health services issues [9], it may be assumed that people felt that the COVID-19 lockdown regulations met their health-related concerns and needs for health safety, contributing to QOL in this domain. The importance of the experience that one’s needs were met may be further supported by the findings that during lockdown, QOL of those who were defined at risk for COVID-19 complications did not differ from those who were not at risk and it was even higher than previously reported in the environment domain. We found that women, young adults, and the unemployed were at high risk for lower QOL, again delineating populations who require health-promoting action. 

Furthermore, we expanded our study toward the investigation of the complex interplay between multiple factors associated with QOL to reveal the most relevant ones, which may serve as protectors and should be a target for public health-promoting action to support QOL. The multivariate analysis detected that factors affecting QOL are psychological distress, number of terminated activities (including leisure activities and employment), the reduced experience of meaning, satisfaction, and enjoyment in available activities; failure to keep daily routines; lower resilience through the personal perception of self-efficacy in coping with a challenging situation, and experience of social and community disconnectedness. Health promotion strategies may be grounded on psychoeducation, providing broad but structured information about the situation and making an explicit bi-directional connection between emergent situations and behaviors that are required to maintain all aspects of health. These strategies were initially provided by “Coping with Stress” guidelines [39] and “Disruption in normal life activities” document [40] but should be further expanded. Actions toward supporting participation in daily life activities may be especially efficient since participation is associated with both QOL and psychological distress and is mediating between them. The latter suggests that health professions, such as occupational therapists, are in a good position to provide public health interventions, contributing to the overall health force that is busy coping with the COVID-19 outbreak. 

The study has several limitations. The data were collected based on self-report with well-known limitations of this methodology. To reach a higher number of participants, we distributed the survey through social networks, which assumes that it was more accessible for specific segments of the healthy population. Still, the number of the participants may be seemed as less representative to draw a conclusion about the impact of the pandemic on the whole population. In addition, the study sample was unbalanced in terms of gender (two-thirds of the participants were women), which may have had an impact on the study results. 

## 5. Conclusions

The COVID-19 pandemic has the great potential for the secondary impact while reducing health-related QOL and evoking psychological distress, disrupting dimensions of participation in daily life activities, and decreasing experiences of connectedness, that all together further affect QOL among the general population. These findings, which are in line with theory on the QOL components and affecting factors, raise a profound concern given the association of aforementioned factors with immediate and long-term parameters of health and well-being. Based on the findings, we strongly suggest further development and delivery of public health strategies, following harbingers in the field, in order to relieve depression and stress caused by the situation, limit disruption in daily activities, expand self-efficacy, the experience of community membership, strategies to keep routines, enjoyment and satisfaction with the activities, and reduce the experience of loneliness. Such public health strategies will diminish the secondary, long-lasting impact of the pandemic, which generates high burdens on people and the whole society. Moreover, the study detects new at-risk populations for the secondary impact who had not received much attention from the health and social services until now in the context of the COVID-19 outbreak: Young adults, women, and the unemployed. These groups have unique characteristics and require focused actions to meet their needs for further reduction of the secondary pandemic impact. Addressing the limitations in participation in daily life activities may be an especially effective strategy in this situation among the general healthy population, as the participation contributes directly to health and QOL and mitigates the impact of psychological distress.

## Figures and Tables

**Table 1:** Analysis of differences in the main study parameters by the demographic variables (*n* = 571).

	Gender	Employment Status	Risk Groups	Communication	Keeping Daily Routines	Age
DASS						
Depression	Z = −2.2 *p* = 0.03	Z = −3.8 *p* = 0.001	Z = −1.77 *p* > 0.05	Z = −0.86 *p* > 0.05	Z = −3.26 *p* = 0.001	H(4) = 22.3 *p* = 0.001
Anxiety	Z = −2.57 *p* = 0.01	Z = −4.1 *p* = 0.001	Z = −0.7 *p* > 0.05	Z = −1.1 *p* > 0.05	Z = −1.08 *p* > 0.05	H(4) = 20.3 *p* = 0.001
Stress	Z = −4.3 *p* = 0.001	Z = −3.98 *p* = 0.001	Z = −3.3 *p* = 0.001	Z = −0.032 *p* >0.05	Z = −2 *p* = 0.042	H(4) = 29.8 *p* = 0.001
UCLA	Z = −1.5 *p* > 0.05	Z = −2.4 *p* = 0.018	Z = −0.35 *p* > 0.05	Z = −0.99 *p* > 0.05	Z = −2.67 *p* = 0.008	H(4) = 1.1 *p* > 0.05
Sense of Community Index						
Reinforcement of needs	Z = −1.9 *p* > 0.05	Z = −0.15 *p* > 0.05	Z = −0.5 *p* > 0.05	Z = −0.17 *p* > 0.05	Z = −0.46 *p* > 0.05	H(4) = 6 *p* > 0.05
Membership	Z = −0.04 *p* > 0.05	Z = −0.18 *p* > 0.05	Z = −0.28 *p* > 0.05	Z = −0.26 *p* > 0.05	Z = −0.37 *p* > 0.05	H(4) = 3.3 *p* > 0.05
Influence	Z = −0.5 *p* > 0.05	Z = −0.46 *p* > 0.05	Z = −0.02 *p* > 0.05	Z = −0.78 *p* > 0.05	Z = −0.04 *p* > 0.05	H(4) = 3.4 *p* > 0.05
Shared Emotional Connection	Z = −1.17 *p* > 0.05	Z = −0.43 *p* > 0.05	Z = −0.57 *p* > 0.05	Z = −0.64 *p* > 0.05	Z = −0.37 *p* > 0.05	H(4) = 3.2 *p* > 0.05
GSE	Z = −1.2 *p* > 0.05	Z = −2.4 *p* = 0.017	Z = −0.79 *p* > 0.05	Z = −1.47 *p* > 0.05	Z = −3.3 *p* = 0.001	H(4) = 6.9 *p* > 0.05
Adults Subjective Assessment of Participation						
Diversity	Z = −2.1 *p* = 0.039	Z = −3.78 *p* = 0.001	Z = −0.68 *p* > 0.05	Z = −0.02 *p* > 0.05	Z = −3.55 *p* = 0.001	H(4) = 23.75 *p* = 0.001
Stopped activities	Z = −2.4 *p* = 0.017	Z = −0.33 *p* > 0.05	Z = −1.9 *p* > 0.05	Z = −0.28 *p* > 0.05	Z = −0.31 *p* > 0.05	H(4) = 30.69 *p* = 0.001
Intensity	t(568) = −1.44 *p* > 0.05	t(569) = 0.09 *p* > 0.05	t(569) = −0.77 *p* > 0.05	t(569) = 1.52 *p* > 0.05	t(569) = −0.59 *p* > 0.05	F(4) = 1.74 *p* > 0.05
Meaning	Z = −3.1 *p* = 0.003	Z = −4.3 *p* = 0.001	Z = −0.69 *p* >0.05	Z = −0.11 *p* >0.05	Z = −3.45 *p* = 0.001	H(4) = 29.3 *p* = 0.001
Enjoyment	t(568) = −0.4 *p* > 0.05	t(569) = 1.65 *p* > 0.05	t(569) = 2 *p* = 0.044	t(569) = 1.73 *p* > 0.05	t(569) = −2.3 *p* = 0.024	F(4) = 4.65 *p* = 0.001
Satisfaction	t(568) = −1.16 *p* > 0.05	t(569) = 2.06, *p* = 0.04	t(569) = −0.57 *p* > 0.05	t(569) = 0.85 *p* > 0.05	t(569) = −2.55 *p* = 0.011	F(4) = 2.11 *p* > 0.05
Activities at home (%)	Z = −3.6 *p* = 0.001	Z = −4.3 *p* = 0.001	Z = −1.19 *p* > 0.05	Z = −0.35 *p* > 0.05	Z = −0.92 *p* > 0.05	H(4) = 21.6 *p* = 0.001
Activities alone (%)	Z = −2.5 *p* = 0.012	Z = −2.84 *p* = 0.01	Z = −2 *p* = 0.041	Z = −2 *p* = 0.045	Z = −0.56 *p* > 0.05	H(4) = 16.85 *p* = 0.002
WHOQOL-BREF						
Physical	Z = −2.28 *p* = 0.024	Z = −6.34 *p* = 0.001	Z = −1.65 *p* > 0.05	Z = −2.54 *p* = 0.011	Z = −2.2 *p* = 0.022	H(4) = 5.4 *p* > 0.05
Psychological	Z = −2.33 *p* = 0.017	Z = −2.78 *p* = 0.005	Z = −0.87 *p* > 0.05	Z = −0.27 *p* > 0.05	Z = −2.5 *p* = 0.013	H(4) = 3.68 *p* > 0.05
Social	Z = −0.015 *p* > 0.05	Z = −2.4 *p* = 0.015	Z = −1.04 *p* > 0.05	Z = −0.13 *p* > 0.05	Z = −3.2 *p* = 0.001	H(4) = 3.4 *p* >0.05
Environmental	Z = −0.81 *p* > 0.05	Z = −3 *p* = 0.003	Z = −0.29 *p* > 0.05	Z = −0.35 *p* > 0.05	Z = −0.73 *p* > 0.05	H(4) = 10 *p* = 0.04

Note: DASS—Depression Anxiety Stress Scale; GSE—General Self Efficacy; UCLA—UCLA Loneliness Scale; WHOQOL-BREF—WHO Quality of Life questioner.

**Table 2:** Correlations between the study variables (*n* = 571).

			WHOQOL-BREF				GSE	DASS			UCLA	Sense of Community Index			
			Physical	Psychological	Social	Environmental		Stress	Anxiety	Depression		Reinforcement of Needs	Membership	Influence	Shared Emot. Connect
GSE		r	0.294 **	0.484 **	0.330 **	0.379 **									
		*p*	0.000	0.000	0.000	0.000									
DASS	Stress	r	−0.49 **	−0.6 **	−0.42 **	−0.44 **	−0.29 **								
		*p*	0.000	0.000	0.000	0.000	0.000								
	Anxiety	r	−0.42 **	−0.53 **	−0.39 **	−0.40 **	−0.27 **	0.73 **							
		*p*	0.000	0.000	0.000	0.000	0.000	0.000							
	Depression	r	−0.49 **	−0.72 **	−0.48 **	−0.49 **	−0.41 **	0.76 **	0.72 **						
		*p*	0.000	0.000	0.000	0.000	0.000	0.000	0.000						
UCLA		r	−0.32 **	−0.51 **	−0.54 **	−0.44 **	−0.39 **	0.43 **	0.44 **	0.56 **					
		*p*	0.000	0.000	0.000	0.000	0.000	0.000	0.000	0.000					
Sense of Community Index	Reinforcement of needs	r	0.14 **	0.22 **	0.22 **	0.23 **	0.18 **	−0.11 *	−0.10 *	−0.17 **	−0.32 **				
		*p*	0.001	0.000	0.000	0.000	0.000	0.012	0.014	0.000	0.000				
	Membership	r	0.11 **	0.2 **	0.16 **	0.21 **	0.16 **	−0.06	−0.05	−0.11 **	−0.21 **	0.82 **			
		*p*	0.008	0.000	0.000	0.000	0.000	0.140	0.228	0.007	0.000	0.000			
	Influence	r	0.11 *	0.12 **	0.11 **	0.17 **	0.11 **	−0.01	−0.01	−0.06	−0.19 **	0.79 **	0.87 **		
		*p*	0.012	0.004	0.009	0.000	0.007	0.895	0.813	0.131	0.000	0.000	0.000		
	Shared Emot. Connect	r	0.10 *	0.13 **	0.14 **	0.17 **	0.11 **	−0.02	−0.02	−0.07	−0.17 **	0.8 **	0.84 **	0.89 **	
		*p*	0.017	0.001	0.001	0.000	0.009	0.669	0.663	0.080	0.000	0.000	0.000	0.000	
Age		r	0.05	0.09 *	0.01	0.16 **	−0.06	−0.23 **	−0.15 **	−0.19 **	−0.02	0.03	0.04	0.01	0.04
		*p*	0.256	0.028	0.824	0.000	0.168	0.000	0.000	0.000	0.729	0.470	0.354	0.809	0.325
Adults Subjective Assessment of Participation	Diversity	r	0.08	0.1 *	0.00	0.08	0.05	0.00	−0.02	−0.07	−0.09 *	0.06	0.05	0.09 *	0.07
		*p*	0.061	0.020	0.992	0.073	0.201	0.983	0.569	0.114	0.035	0.176	0.275	0.035	0.103
	Stopped activities	r	−0.09 *	−0.03	0.02	−0.08 *	0.07	0.08	0.12 **	0.03	0.01	0.16 **	0.16 **	0.16 **	0.14 **
		*p*	0.041	0.499	0.729	0.046	0.08	0.061	0.003	0.552	0.768	0.000	0.000	0.000	0.001
	Intensity	r	0.05	0.08	0.10 *	0.02	0.16 **	0.00	0.01	−0.05	−0.08	0.07	0.08	0.05	0.09 *
		*p*	0.212	0.058	0.013	0.700	0.000	0.992	0.853	0.238	0.060	0.089	0.063	0.285	0.042
	Meaning	r	0.1 *	0.17 **	0.1 *	0.1*	0.18 **	0.00	−0.02	−0.11 **	−0.2 **	0.13**	0.12 **	0.14 **	0.14 **
		*p*	0.018	0.000	0.013	0.015	0.000	0.967	0.689	0.007	0.000	0.002	0.006	0.001	0.001
	Enjoyment	r	0.25 **	0.31 **	0.25 **	0.16 **	0.22 **	−0.14 **	−0.12 **	−0.21 **	−0.21 **	0.21 **	0.17 **	0.15 **	0.18 **
		*p*	0.000	0.000	0.000	0.000	0.000	0.001	0.004	0.000	0.000	0.000	0.000	0.000	0.000
	Satisfaction	r	0.27 **	0.37 **	0.37 **	0.23 **	0.28 **	−0.22 **	−0.22 **	−0.29 **	−0.35 **	0.17 **	0.14 **	0.1 *	0.14 **
		*p*	0.000	0.000	0.000	0.000	0.000	0.000	0.000	0.000	0.000	0.000	0.002	0.012	0.001
	Activities at home (%)	r	−0.1 *	−0.06	0.04	−0.07	0.02	0.09 *	0.04	0.05	0.02	−0.06	−0.06	−0.02	−0.07
		*p*	0.016	0.152	0.384	0.079	0.567	0.043	0.309	0.208	0.634	0.155	0.175	0.569	0.085
	Activities alone (%)	r	−0.07	−0.03	−0.13 **	−0.04	−0.03	−0.03	0.02	0.06	0.11 **	−0.1 *	−0.04	−0.09 *	−0.05
		*p*	0.097	0.466	0.001	0.301	0.502	0.508	0.612	0.170	0.006	0.015	0.312	0.026	0.216

Note: * *p* < 0.05; ** *p* < 0.001; DASS—Depression Anxiety Stress Scale; GSE—General Self Efficacy; UCLA—UCLA Loneliness Scale; WHOQOL-BREF—WHO Quality of Life questioner.

**Table 3:** Regression analysis coefficients (*n* = 751) ^a^.

WHOQOL-BREF Domains	B	St Error B	β	*p* Value
**Physical**				
DASS-Depression	−0.422	0.118	−0.201	0.000
DASS-Stress	−0.491	0.093	−0.285	0.000
ASAP-Enjoyment	4.149	1.091	0.138	0.000
ASAP-Number of stopped activities	−0.365	0.132	−0.098	0.006
General Self Efficacy	3.015	1.169	0.100	0.010
SCI-Influence subscale	0.268	0.136	0.070	0.049
**Psychological**				
DASS-Depression	−0.867	0.078	−0.489	0.000
DASS-Stress	−0.199	0.060	−0.136	0.001
ASAP-Satisfaction	3.558	0.729	0.140	0.000
General Self Efficacy Scale	4.941	0.773	0.193	0.000
SCI-Membership subscale	0.268	0.093	0.079	0.004
**Social**				
DASS-Depression	−0.152	0.066	−0.129	0.022
DASS-Stress	−0.142	0.050	−0.146	0.005
UCLA Loneliness scale	−0.381	0.048	−0.330	0.000
ASAP-Satisfaction	3.221	0.602	0.190	0.000
ASAP-Percent of activities done along	−0.039	0.014	−0.092	0.006
**Environment**				
DASS-Depression	−0.326	0.121	−0.158	0.007
DASS-Stress	−0.233	0.090	−0.137	0.010
General Self Efficacy	6.040	1.154	0.203	0.000
ASAP-Number of stopped activities	−0.492	0.131	−0.134	0.000
SCI-Membership subscale	0.481	0.139	0.122	0.001
UCLA Loneliness scale	−0.373	0.086	−0.185	0.000
Age	0.163	0.048	0.124	0.001

Note: ^a^—Only the last step of each regression model are presented; WHOQOL-BREF—WHO Quality of Life questioner; DASS—The Depression, Anxiety and Stress Scale; ASAP—The Adults Subjective Assessment of participation; SCI—Sense of Community Index.

**Table 4:** Mediating effect of participation dimensions on the WHOQOL-BREF domains (*n* = 571).

	Mediator	Regression Weight	SE	*p*
Physical	Number of stopped activities			
	DASS-Stress	−0.016	0.01	0.015
	GSE	−0.321	0.164	0.048
	SCI-Influence	−0.051	0.026	0.004
	Enjoyment with participation			
	DASS-Depression	−0.042	0.018	0.004
	GSE	0.646	0.274	0.002
	SCI-Influence	0.068	0.03	0.001
Psychological	Satisfaction with participation			
	DASS-Depression	−0.048	0.018	0.001
	GSE	0.651	0.213	0.001
	SCI-Membership	0.034	0.02	0.06
Social relationship	Satisfaction with participation			
	DASS-Depression	−0.028	0.013	0.013
	UCLA Loneliness Scale	−0.059	0.015	0.001
	Percent of activities done along			
	DASS-Depression	−0.013	0.009	0.046
	DASS-Stress	0.015	0.008	0.005
	UCLA Loneliness Scale	−0.013	0.007	0.01
Environment	Number of stopped activities			
	DASS-Stress	−0.037	0.015	0.001
	GSE	−0.422	0.205	0.008
	SCI-Membership	−0.072	0.033	0.001
	age	−0.038	0.012	0.001

Notes: WHOQOL-BREF—WHO Quality of Life questioner; DASS—Depression Anxiety and Stress scale; GES—General Self-Efficacy Scale; SCI—Sense of Community Index.

## Data Availability

The data will be available from the corresponding author upon request.
